# Prof. Dowler and the Medico Chirurgical Review

**Published:** 1852-03

**Authors:** 


ARTICLE VII.

*Prof. Dowler and the Medico Chirurgical Review.*

[It is exceedingly gratifying to see the following notice of our distinguished countryman, Prof. Bennett Dowler of New Orleans, in the leading European Medical Review. It speaks well for the ability and correctness of observation of Dr. Dowler, and for the independence of the Review itself.]

"Dr. Dowler has made himself conspicuous among his brethren, by his refusal to receive certain of those *Neurological** *doctrines, which, under one form or another, are now generally admitted among well informed physiologists.* We do not quarrel with him for declining to accept *the double system of excito-motor and of sensori-volitional nerves*, SUCH HAVING, AS WE NOW BELIEVE, NO REAL EXISTENCE IN NATURE ; and we have a strong sympathy with his objection to the new terms—diastalic, esodic, exodic, anodic, cathodic, paltodic, anastalic, catastaltic, peristaltic, &c., by the adoption of which, we venture to think,

---

* The Italics and caps are ours.

a comparatively easy subject would be rendered obscure. * * * * Dr. Dowler has a fine field for experiment, being able to procure alligators for purposes, for which European physiologists must content themselves with frogs; and the former animals not only exhibiting the phenomena of reflex action upon a much larger scale, but also possessing a most extraordinary tenacity of life. His accounts of his experiments are very graphically drawn," &c.

[The following remarks upon the above quotation, by the editor of the New Orleans Medical and Surgical Journal, so fully coincide with our own views, and so clearly set forth Dr. Dowler's position, that we insert them in lieu of further comment.]

"We have not space to copy the whole Review, nor to notice certain details, in which the Reviewer dissents from Dr. Dowler, particularly in the concluding query, from which it appears that the Reviewer mistakingly supposes that Dr. Dowler wishes to establish an independent *Me Ego*, or conscious mind, in the centre of each division of a vivisected or divided animal. Dr. D's. argument is in favor of a diffused, and not in favor of a central sensorium, in the entire, much less in the divided animal. The Reviewer, probably, has not seen the whole series of Dr. D's. papers on this subject, or he would not have attributed so much to *automatic action* in the phenomena detailed by Dr. D., who has thoroughly examined and fully refuted this mechanical idea, as applied to explain the varied intelligential actions of decapitated and divided animals, as related in his numerous papers.

The readers of this Journal will probably be not less surprised than ourself, at the moral courage manifested by the most eminent Medical Review extant, in abjuring all faith in the Four-fold Nervous System, "*now generally admitted*," (to use its own words) "*amongst well informed physiologists, such having, as we now believe*, NO REAL EXISTENCE IN NATURE.'

Such an admission forms an epoch in the mighty stream of knowledge, that has so long flowed in the Medico Chirugical Review, and is a luminous exemplification of an editorial principle announced in the June (1820) number of that Journal, more than thirty-one years ago, namely, "This journal is free and independent as the air we breathe; we trust it never will be *deterred* from doing that which is right, or *seduced* to do that which is wrong."

"The double system of sensori-volitional nerves," which the illustrious Bell was supposed to have discovered, found in this Review its mightiest and earliest advocate. In a Review of Mr. Bell's experiments, on the Nerves, in March, 1822, the Reviewer says:

"'No man in this country works harder in unraveling those mysteries of the nervous system which puzzle our senses, than the present distinguished teacher in the venerable school of the Hunters.

"Some people may ask, to what does this discussion lead, after all? It may be answered, that both the surgeon and physician are interested in knowing, that *two sets of nerves* are distributed to the face, having *distinct functions.*

"'When the air-balloon was first discovered, some one flippantly asked Doctor Franklin, what was the use of it? The Doctor answered, in the Socratic manner, by asking another question—'*what is the use of a new-born infant?—it may become a man.*'"

We repeat that this admission forms an epoch in scientific progress, because with certain individuals it will weigh more than any amount of *demonstration, intuition*, or possibly *revelation itself.*"

